# Degradation of aqueous synthesized CdTe/ZnS quantum dots in mice: differential blood kinetics and biodistribution of cadmium and tellurium

**DOI:** 10.1186/1743-8977-10-37

**Published:** 2013-08-06

**Authors:** Na Liu, Ying Mu, Yi Chen, Hubo Sun, Sihai Han, Mengmeng Wang, Hui Wang, Yanbo Li, Qian Xu, Peili Huang, Zhiwei Sun

**Affiliations:** 1 Department of Toxicology and Sanitary Chemistry, School of Public Health, Capital Medical University, No.10 Xitoutiao You An Men, Beijing 100069, China; 2 Beijing Key Laboratory of Environmental Toxicology, Capital Medical University, No.10 Xitoutiao You An Men, Beijing 100069, China; 3 Research Center for Analytical Instrumentation, Institute of Cyber-Systems and Control, State Key Lab. of Industrial Control Technology, Zhejiang University, 866 Yuhangtang Road, Hangzhou 310058, China; 4 Basic Medical Experimental Teaching Center, Capital Medical University, No.10 Xitoutiao You An Men, Beijing 100069, China; 5 College of Food and Bioengineering, Henan University of Science and Technology, Kaiyuan Ave 263, Luoyang 471023, China; 6 Ministry of Education Key Laboratory of Environmental Medicine and Engineering, Southeast University, No.87 Dingjiaqiao, Nanjing 210009, China

**Keywords:** Biodistribution, Chemical fate, Degradation, Intravenous, ICP-MS, Kinetics, Mice, Nanoparticles, Quantum dots

## Abstract

**Background:**

Quantum dots (QDs) have been used as novel fluorescent nanoprobes for various bioapplications. The degradation of QDs, and consequent release of free cadmium ions, have been suggested to be the causes of their overall toxicity. However, in contrast to sufficient investigations regarding the biological fate of QDs, a paucity of studies have reported their chemical fate *in vivo*. Therefore, the overall aim of our study was to understand the chemical fate of QDs *in vivo* and explore analytical techniques or methods that could be used to define the chemical fate of QDs *in vivo*.

**Methods:**

Male ICR mice were administered a single intravenous dose (0.2 μmol/kg) of aqueous synthesized CdTe/ZnS aqQDs. Inductively coupled plasma-mass spectrometry (ICP-MS) was used to simultaneously measure the concentrations of cadmium (Cd) and tellurium (Te) in the blood and tissues over the course of a 28 day period. We compared the blood kinetic parameters and biodistributions of Cd and Te, and used the molar ratio of Cd:Te as a marker for QDs degradation.

**Results:**

Cd and Te display different blood kinetics and biodistribution profiles. The Cd:Te ratio in the blood did not vary significantly within the first hour compared with intact CdTe/ZnS aqQDs. The Cd:Te ratio decreased gradually over time from the 6 h time point on. Cd accumulated in the liver, kidneys, and spleen. Te was distributed primarily to the kidneys. Sharp time-dependent increases in the Cd:Te ratio were found in liver tissues.

**Conclusions:**

QDs can undergo degradation *in vivo*. *In vitro*, QDs are chemically stable and do not elicit the same biological responses or consequences as they do *in vivo*. Our methods might provide valuable information regarding the degradation of QDs *in vivo* and may enable the design and development of QDs for biological and biomedical applications.

## Background

Quantum dots (QDs) are recognized to be novel, high-performance biological probes that are at the forefront of nano-biotechnology research because they possess many attractive optical properties, including a high photoluminescent quantum yield (PLQY), a broad absorption spectrum coupled with narrow emission, and strong photostability [1-3]. However, cadmium (Cd), which is capable of inducing known toxicities in humans (including hepatic, renal, neurologic, and/or genetic toxicities) [4,5], is the most abundant component of QDs. The degradation of QDs, and consequent release of free cadmium ions (Cd^2+^), have been suggested to be the causes of their overall toxicity. Therefore, it is critically important to systematically evaluate the chemical fate of QDs given their widespread applications in biology [6].

Two strategies were used in order to synthesize QDs: organic synthesis (orQDs) and aqueous synthesis (aqQDs). Aqueous synthetic strategies are simpler, cheaper, and more environmentally friendly than organic synthesis methods. More importantly, aqQDs are naturally water-dispersed and do not have large amounts of hydrophilic ligands covering their surfaces. Therefore, aqQDs possess a much smaller hydrodynamic diameter than orQDs. Highly luminescent aqQDs have been thoroughly developed and used in various bioapplications, including bioimaging and protein chips [7-9]. Cadmium-telluride (CdTe) QDs surrounded by a thin shell or cap of zinc sulfides (ZnS) (CdTe/ZnS aqQDs) are one of the most successful examples. As with all QDs, the CdTe core of the CdTe/ZnS aqQDs is composed of covalently bound Cd and tellurium (Te) that are held at a constant molar ratio (Cd:Te). As a result of the stability of the covalently bound CdTe core and the apparent ‘inertness’ of the CdTe complex (as opposed to the reactive nature of Cd^2+^), this bound Cd complex has no valence charge and, therefore, no biological reactivity. The exposed CdTe/ZnS aqQDs are therefore believed by many to be biologically safe and non-toxic. However, if the CdTe core is degraded, the release of Cd^2+^ and telluride ions (Te^2-^) from the CdTe core will increase the risk of target organ toxicities [4,5,10].

To better understand and predict the efficacy and side effects of QDs *in vivo*, four key questions need to be addressed: What is the pharmacokinetic profile of QDs? Where are these nanocrystals deposited (tissue disposition of QDs) within the body? How long do the deposited QDs remain in the tissues or organs? What happens to the QDs particles when they are in a biological system? Work conducted by Fischer’s group resulted in the first quantitative report on the *in vivo* biodistribution of QDs in 2006 [11]. Several subsequent *in vivo* pharmacokinetic studies of QDs have been completed [12-14]. The results of these studies suggest several key points: (i) administered QDs have a rather wide range (from 48 min to 20 h) of half-lives (*t*_*1*/*2*_) in the bloodstream; (ii) QDs do not remain in the circulation and tend to accumulate in organs and tissues; (iii) QDs that are injected intravenously are more likely to accumulate in the liver, spleen, and kidneys; and (iv) the *in vivo* behavior of QDs is greatly dependent on their hydrodynamic diameters. QDs with smaller hydrodynamic diameters are more rapidly and efficiently eliminated via renal clearance in mice than those with large hydrodynamic diameters (>15 nm). In contrast to the sufficient investigations outlined above, there have been relatively few studies addressing the chemical fate of QDs *in vivo*.

In our previous studies regarding the pharmacokinetics and tissue distribution of CdTe aqQDs, we found that Cd and Te display different plasma kinetics and distribution patterns [15]. The different plasma kinetics and distribution patterns of Cd and Te imply that CdTe aqQDs may undergo degradation *in vivo*. To further explore the degradation of QDs, we investigated the *in vivo* properties of CdTe/ZnS aqQDs, including blood pharmacokinetics and the long-term biodistribution of Cd and Te. Moreover, based on the atomic weights of Cd and Te, the Cd:Te ratios in the blood and tissues over time were calculated and were used to reflect the general stability and conditions of CdTe/ZnS aqQDs in biological systems. As compared with the initial and normal Cd:Te ratio in CdTe/ZnS aqQDs, steady or unchanged Cd:Te ratios in the blood and tissues over time indicate that the CdTe/ZnS aqQD complexes have remained intact. In contrast, alterations in the Cd:Te ratio signify disintegration of the complex. In addition, the chemical fate of CdTe/ZnS aqQDs *in vitro* and *in vivo* were examined as well. Based on the blood kinetic parameters and biodistributions of Cd and Te, as well as alterations in the Cd:Te ratio, we believe that we can assess the chemical fate of CdTe/ZnS aqQDs in biological systems. Although QDs have different core compositions (for example, gallium-, copper-, lead-, and arsenide-based QDs), different QDs may behave similarly in biological systems. The information generated from our studies may contribute to the general understanding of QDs and the evaluation of the biological risks associated with their use.

## Results

### Characteristics of CdTe/ZnS aqQDs

**Figure 1:**
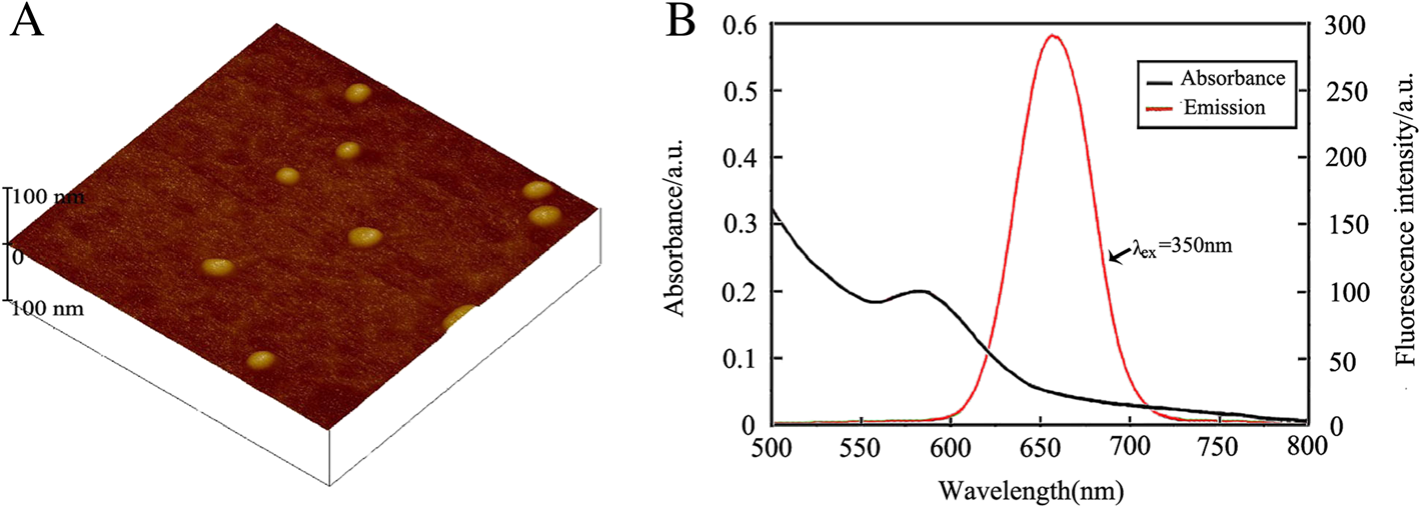
**Characteristics of CdTe/ZnS aqQDs: (A) AFM image, (B) absorption and emission spectra.** The average size was 19.3 ± 2.2 nm in diameter. The maximal emission was observed at approximately 652 nm following excitation at 350 nm.

For atomic force microscopy (AFM) measurements, only the Z-dimension was used to determine the size in order to avoid probe-related artifacts. These measurements yielded a mean size of 19.3 ± 2.2 nm. The shape of the CdTe/ZnS aqQDs was approximately spherical (Figure 1A). The emission spectra of the aqQDs are presented in Figure 1B. The maximal emission was 652 nm (at λex = 350 nm). The concentration of the CdTe/ZnS aqQDs stock solution was 2.5 μmol/ml (calculated based on the molar mass of Cd). The Cd:Te ratio was 3:1, and the molar ratio of zinc (Zn) to Cd (Zn:Cd) was 1:1.

### Stability of CdTe/ZnS aqQDs *in vitro*

**Figure 2:**
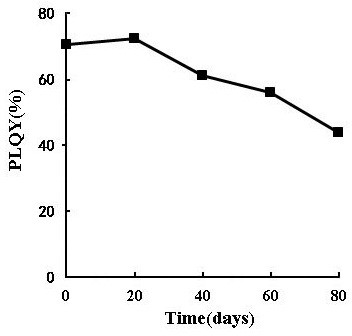
**PLQY transformation of CdTe/ZnS aqQDs *in situ *(pH 8.3) for periods up to 80 days.**

The stability data for the CdTe/ZnS aqQDs *in vitro* are shown in Figure 2 and Table 1. Figure 2 illustrates that in the first 20 days, the PLQYs of CdTe/ZnS aqQDs *in situ* were not significantly different (ranging from 70.3 to 72.3%). The PLQYs gradually decreased over the next 20 days, and the values had dropped to 43.8% of their original values 80 days later. Table 1 shows that the maximal emission (652 nm) of CdTe/ZnS aqQDs was not altered by dialyses lasting up to 3 d in pH 7.4 buffered solutions, but dialysis rapidly reduced the relative fluorescence intensity after 6 h (from 271.0 to 144.1). After 3 d, only 55.2 remained. In the filtrate, the concentrations of Cd, Te, and Zn gradually increased with time, but Cd:Te ratios did not vary significantly (the molar ratio of Cd and Te in CdTe/ZnS aqQDs used in this study was 3:1). Zn:Cd ratios were not significantly different (the molar ratio of Zn and Cd in CdTe/ZnS aqQDs used in this study was 1:1) in the first 6 h, but at the 24 h, 2 and 3 d time-points, the ratios had become slightly elevated (from 1.13:1 to 1.26:1).

**Table 1:** Stability of CdTe/ZnS aqQDs in buffer solutions mimicking blood (pH 7.4)

**Time**	**Maximal emission (nm)**	**Relative fluorescent unit**	**Metal concentrations in filtrate (ng/ml)**			**Molar ratios of metals in filtrate**	
			**Cd**	**Te**	**Zn**	**Cd:Te**	**Zn:Cd**
Initial time	652	271.0 ± 6.5	ND	ND	ND	ND	ND
15 min	652	270.5 ± 5.8	2.08 ± 0.12	0.81 ± 0.01	1.30 ± 0.01	2.92:1	1.08:1
30 min	652	270.6 ± 7.9	7.20 ± 0.21	2.90 ± 0.02	4.08 ± 0.05	2.82:1	0.98:1
1 h	652	270.0 ± 9.0	7.72 ± 0.48	2.88 ± 0.30	4.82 ± 0.12	3.05:1	1.08:1
6 h	652	144.1 ± 12.8	8.52 ± 0.95	3.36 ± 0.45	5.02 ± 0.38	2.88:1	1.02:1
24 h	652	104.2 ± 11.1	11.90 ± 1.06	4.91 ± 0.36	7.77 ± 1.01	2.75:1	1.13:1
2 d	652	86.2 ± 10.9	12.87 ± 1.24	5.04 ± 0.44	9.08 ± 0.91	2.90:1	1.22:1
3 d	652	55.2 ± 2.6	14.42 ± 1.80	5.63 ± 0.61	10.50 ± 1.08	2.91:1	1.26:1

*ND* not detectable. All data are represented as the mean ± SD, n = 3.

### Comparative Cd and Te blood kinetics

We did not observe significant changes in body or organ weights in the mice after tail vein injections of CdTe/ZnS aqQDs (Additional file 1: Table S1). Additionally, no symptoms of illness were observed in the mice at the dose of aqQDs administered during the experimental period, and all of the animals survived until the end of the experimental period.

The Cd and Te concentrations in the blood are presented in Table 2. Table 2 shows that blood Cd concentrations were much higher than Te concentrations for the first day after injection. At 3 d post-injection, the Cd concentrations were below the limit of detection and were much lower than the Te concentrations. The Cd:Te ratios did not vary significantly in the first hour. The ratios were 3.01:1 (1 min), 2.95:1 (15 min), 2.95:1 (30 min) and 2.94:1 (1 h). All of the ratios were approximately 3:1, which corresponds to the Cd:Te ratio in the CdTe/ZnS aqQDs used in this study. From the 6 h time point on, the Cd:Te ratios decreased gradually over time from 2.48:1 to 1.87:1. The main kinetic parameters shown in Table 3 were fit using a two-compartment model with a weighting factor of 1/c^2^, in which Cd and Te varied. Cd exhibited a significantly greater area under the blood concentration-time profiles (AUC) (2.16 ± 0.07 μg · h/ml vs. 0.99 ± 0.13 μg · h/ml, *p* < 0.01), clearance (CL) (10.40 ± 0.48 ml/h/kg vs. 8.99 ± 1.53 ml/h/kg, *p* < 0.05) and a rapider elimination half-life (*t*_*1*/*2β*_) (12.41 ± 0.26 h vs. 14.40 ± 1.33 h, *p* < 0.05) than Te.

**Table 2:** **Blood concentration**-**time curves of Cd and Te in mice exposed to CdTe**/**ZnS aqQDs**

**Exposed time**	**Cd (ng/ml)**	**Te (ng/ml)**	**Cd:Te**
1 min	195.23 ± 1.78	73.60 ± 2.40	3.01:1
15 min	142.40 ± 3.40	54.80 ± 0.57	2.95:1
30 min	123.70 ± 2.68	47.60 ± 3.32	2.95:1
1 h	108.80 ± 1.98	42.05 ± 1.13	2.94:1
6 h	91.00 ± 2.24	41.70 ± 3.11	2.48:1
12 h	66.23 ± 1.90	26.50 ± 1.27	2.30:1
24 h	24.33 ± 4.29	10.10 ± 4.25	1.87:1
3 d	ND	7.10 ± 1.42	ND

*ND* not detectable. All data are represented as the mean ± SD, n = 6.

**Table 3:** **Kinetic parameters of Cd and Te in mice exposed to CdTe**/**ZnS aqQDs**

**Kinetic parameters**	**Unit**	**Cd**	**Te**
Vd	ml/kg	110.48 ± 1.36	115.91 ± 2.44
AUC	μg · h /ml	2.16 ± 0.07^**^	0.99 ± 0.13
CL	ml/h/kg	10.40 ± 0.48^*^	8.99 ± 1.53
*t*_*1*/*2α*_	h	0.14 ± 0.06	0.13 ± 0.03
*t*_*1*/*2β*_	h	12.41 ± 0.26^*^	14.40 ± 1.33

Vd, apparent volume of distribution, AUC, area under the blood concentration-time profiles, CL, clearance *t*_*1*/*2α*_, distribution half-life, *t*_*1*/*2β*_, elimination half-life.

Kinetic parameters (AUC, CL and *t*_*1*/*2β*_) of Cd were significantly different compared with those of Te in the blood, ^*^p < 0.05, ^**^p < 0.01.

All data are represented as the mean ± SD, n = 6.

### Comparative Cd and Te tissue distributions

**Figure 3:**
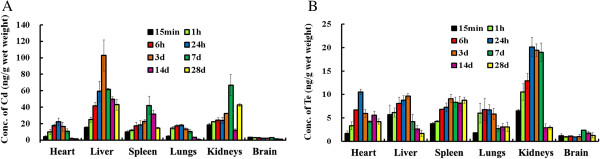
**Bio-distribution of Cd (A) and Te (B) in mice exposed to CdTe/ZnS aqQDs.** ICR mice were injected via tail vein with CdTe/ZnS aqQDs (0.2 μmol/kg). Serial sacrifices were carried out at 15 min, 1 h, 6 h, 24 h, 3 d, 7 d, 14 d, and 28 d after dosing. Several organs/tissues, including heart, liver, spleen, lungs, kidneys, and brain were isolated to determine concentrations of Cd and Te with ICP-MS. The mean values of the control were deducted as the baseline (Additional file 2: Table S2). All data are represented as the mean ± SD, n = 6.

Figure 3A illustrates the tissue distribution of Cd in ICR mice exposed to CdTe/ZnS aqQDs. After intravenous injection, Cd mainly accumulated in the liver and kidneys. Accumulation was also observed in the spleen, lungs and heart. Cd was rarely distributed in the brain. The peak concentration and peak time of Cd in each organ after administration differed. They were: 22.1 ± 4.74 ng/g at 24 h in the heart, 102.82 ± 18.93 ng/g at 3 d in the liver, 42.04 ± 11.22 ng/g at 7 d in the spleen, 18.03 ± 1.27 ng/g at 24 h in the lungs and 66.75 ± 12.75 ng/g at 7 d in the kidneys. By plotting the tissue concentrations versus time, we found that Cd elimination from the liver, kidneys and spleen was significantly slower than Cd elimination from the lungs and heart. Moreover, there were obvious increases in the Cd concentrations in the kidneys at 28 d. At 28 d post-exposure, 42.94, 14.69 and 42.60 ng/g remained in the liver, spleen and kidneys.

The distribution of Te in different tissues is shown in Figure 3B. The kidneys appeared to be the major target organs for Te deposition. Te was also deposited in the heart, liver, spleen and lungs. The brain contained only a very small amount of Te. The peak concentration and peak time of Te in each organ after administration differed. They were: 10.55 ± 0.58 ng/g at 24 h in the heart, 9.63 ± 0.56 ng/g at 3 d in the liver, 9.10 ± 0.87 ng/g at 3 d in the spleen, 6.74 ± 1.07 ng/g at 24 h in the lungs and 20.10 ± 2.09 ng/g at 24 h in the kidneys. The peak times of Te in the heart, liver and lungs after administration were similar to Cd in these organs. In the spleen, Te concentrations increased during the first 3 d and reached peak concentrations (9.10 ng/g). However, from 3 to 28 d, the Te concentrations did not vary significantly (9.10–8.13 ng/g), indicating that elimination had rarely occurred. In the kidneys, the Te concentrations increased sharply during the first day (from 6.53 to 20.10 ng/g), but elimination was significantly slower from 24 h (20.10 ng/g) to 7 d (19.02 ng/g). At 14 d post-exposure, the majority of Te had been eliminated, with only 2.89 ng/g remaining.

### Time-dependent changes in the Cd:Te ratios

The Cd:Te ratios in the tissues were calculated over time and are shown in Table 4. At the 15 min time-point, they were: 2.77:1 (heart), 3.02:1 (liver), 3.02:1 (spleen), 2.94:1 (lungs), 3.21:1 (kidneys), and 3.22:1 (brain). All of the ratios were approximately 3:1, which corresponds to the molar ratio of Cd and Te in the CdTe/ZnS aqQDs used in this study. Furthermore, different trends in the Cd:Te ratios in the organs were observed. The Cd:Te ratios in the heart did not differ significantly over a period of 7 days, but began to decrease gradually after 14 d. In the liver, the Cd:Te ratios increased sharply with time from 1 h through 28 d. In the spleen and lungs, the Cd:Te ratios did not change significantly over a period of 3 d, increased at 7 d and decreased from 14 d on. In the kidneys, the Cd:Te ratios decreased at 24 h and increased gradually from 7 d on. These findings indicate that the CdTe/ZnS aqQDs steadily disintegrated *in vivo*. In addition, the degree of degradation of CdTe/ZnS aqQDs differed in various organs.

**Table 4:** **Cd**:**Te radios in the tissues of mice exposed to CdTe**/**ZnS aqQDs**

**Exposed time**	**Heart**	**Liver**	**Spleen**	**Lungs**	**Kidneys**	**Brain**
15 min	2.77:1	3.02:1	3.02:1	2.94:1	3.21:1	3.22:1
1 h	3.34:1	4.60:1	3.19:1	2.76:1	2.14:1	3.35:1
6 h	3.03:1	5.86:1	2.88:1	2.91:1	2.41:1	2.81:1
24 h	2.39:1	7.73:1	2.88:1	3.04:1	1.35:1	2.59:1
3 d	3.14:1	12.12:1	2.82:1	2.59:1	1.87:1	2.35:1
7 d	2.93:1	16.85:1	5.72:1	4.31:1	3.98:1	1.37:1
14 d	0.41:1	21.42:1	4.38:1	1.18:1	4.66:1	0.87:1
28 d	0.39:1	29.05:1	1.90:1	0.31:1	16.56:1	1.02:1

## Discussion

This study is an extension of our previous study, which was aimed at understanding the chemical fate of QDs *in vivo*. While there has been no lack of studies regarding the fate and biological safety of QDs, many of these studies were conducted *in vitro*[16-21]. Because cultured cells do not metabolize complex chemicals, *in vitro* cultures can not replicate complicated *in vivo* systems. Therefore, studies examining the actual chemical fate of QDs in intact biological systems (animal studies) are needed. This information is critical for the evaluation of the toxicity of QDs and other nanoparticles.

QDs exhibit narrow emission profiles and can emit clear and bright colors based on their strong fluorescence intensities. A fluorescence spectrometer was used to reveal some of the parameters (size and concentration) of QDs. When QDs degrade (loss of surface atoms and decreases in the size of QDs), blue-shifts in the excitation fluorescence spectra and decreases in the quantum yield are observed [22]. However, using fluorescence intensity to quantify QDs in blood and tissues has been deemed to be problematic due to the high and variable background fluorescence that result from native blood and tissues. Moreover, the fluorescence of QDs is susceptible to environmental factors. For example, the PLQY has been demonstrated to change drastically as a result of surface chemistry, rearrangement of surface ligands, photoenhanced oxidization, and solvent effects. Any of these parameters can potentially cause large deviations in fluorescence measurements [13]. Therefore, using fluorescence intensity and peak wavelength in order to determine the chemical fate of QDs *in vivo* was deemed to be problematic. Inductively coupled plasma-atomic emission spectrometer (ICP-AES) and inductively coupled plasma-mass spectrometry (ICP-MS) are highly sensitive methods that can be used to detect most elements. For example, the detection limit for Cd can approach 0.1 ng/ml using ICP-MS. Fischer et al. [11] first tracked QDs via Cd measurements in the blood and organs of rats using ICP-AES and presented a quantitative report on the kinetic parameters and biodistribution of QDs in the rat. Several subsequent biological fate studies of QDs *in vivo* using ICP-AES and ICP-MS have been reported [12-14]. However, the ICP-AES or ICP-MS analytical techniques were unable to distinguish whether Cd was bound within the QDs or had been released into the tissues. This distinction is important because bound Cd has no valence charge and, thus, is most likely much less biologically reactive or toxic than highly charged free Cd^2+^.

In the work conducted by Fischer’s group [11], QDs were incubated with whole blood, and samples were retrieved and centrifuged at various times in order to investigate whether the QDs interacted with blood components. The supernatant (plasma) was analyzed for QDs using fluorescence spectroscopy and ICP-AES, whereas the pellet was further analyzed using fluorescence microscopy. The authors verified using optical microscopy that the QDs exhibited minimal, nonspecific binding to the cellular blood components of the rat (e.g., erythrocytes). Other studies [13] mimicked the pHs of blood (pH 7.4) and the renal tubules (pH 4.8) in order to investigate the stability of CdSeS/SiO_2_ QDs that had been stored in a buffer for five days. The results revealed that the maximal emission of CdSeS/SiO_2_ QDs was not altered and that the fluorescence intensity was stable after five days of incubation in either pH 7.4 or pH 4.8 buffer solutions. Because the concentration of Cd in the supernatant of the experimental solution in both pH environments did not significantly differ from that in the phosphate buffered saline (PBS) blank, the authors concluded that Cd concentrations could be used to estimate the concentration of QDs in their *in vivo* studies. However, *in vivo* toxicity (or perceived toxicity) of the high surface area-to-volume ratio of QDs, which provides a large available surface for enzymatic degradation and the release of metallic ions, has been previously reported. Fitzpatrick et al. [23] studied the persistence of CdSe/ZnS QDs that had been coated with mPEG 5000 (emitting at 655 nm) in mice. They noted that immediately after injection into the tail vein, emissions related to the QDs were observed in the circulatory system and various organs, including the liver, spleen, lymph and bone marrow. The fluorescence signals observed were found to have been blue-shifted from the initial emission wavelengths, due to the loss of one monolayer of the nanoparticle. Within five days, the emission observed in the liver had faded, indicating that extreme degradation of the QDs had occurred with concomitant release of Cd. Pi et al. [24] labeled mouse embryonic stem cells (ESCs) with QDs and monitored QD-labeling using fluorescence microscopy for 72 h. A rapid loss of QD-labeling in ESCs was observed within 48 h. Transmission electron microscopy (TEM) analysis showed a dramatic decrease in QDs in the vesicles of ESCs at 24 h post-labeling, suggesting that the QDs may have degraded.

Here, we used fluorescence spectrometer and ICP-MS to investigate the chemical fate of CdTe/ZnS aqQDs that had been stored in a buffer (pH 7.4) for 3 days (Table 1). The experimental data indicated that the maximal emission was not altered. The concentrations of Cd, Te and Zn ions released from the CdTe/ZnS QDs gradually increased over time, but were extremely low, and the ratios of Cd:Te and Zn:Cd did not vary significantly. This suggests that the size and composition of CdTe/ZnS aqQDs stored in buffer did not differ significantly from those kept *in situ*[22,23]. On the other hand, the fluorescent intensity was reduced after a 6 h incubation in a pH 7.4 buffer solution. This suggests that the nanocrystals of CdTe/ZnS aqQDs formed surface defects in a pH 7.4 environment.

Next, the Cd and Te concentrations in the blood were measured using ICP-MS, the Cd:Te ratios were calculated, and Cd and Te blood kinetic analyses were conducted. As described in Tables 2 and 3, we observed differences in the Cd:Te ratios and Cd and Te blood kinetic parameters, suggesting that CdTe/ZnS aqQDs break down in the blood. If CdTe/ZnS aqQDs are chemically stable in the blood, steady or unchanged Cd:Te ratios in the blood over time would result. Furthermore, the blood kinetic parameters, CL and *t*_*1*/*2β*_ of Cd and Te would be similar. As shown in Tables 1 and 2, the relative fluorescent intensity of QDs in pH 7.4 buffer solution was reduced rapidly after 6 h. The Cd:Te ratios also varied significantly after 6 h in the blood. These findings indicate that the stability of CdTe/ZnS aqQDs in the blood of the mice is comparable to the stability in buffer solutions (pH 7.4) [13]. QDs exhibited minimal, nonspecific binding to the cellular components of mice blood [11].

Lastly, we examined the uptake of CdTe/ZnS aqQDs in various organs after leaving the bloodstream using ICP-MS. Contrary to their relatively transient fate in the blood, Cd and Te are preferentially distributed into organs and tissues (Figure 3A and B). When the specific tissue concentrations of Cd and Te were examined, Cd was observed to mainly accumulate in the liver and kidneys, which was in agreement with previous reports [13-15,25]. Unlike Cd, the kidneys appeared to be the major target organ for Te deposits. No significant changes in Te accumulation were observed in the heart, liver, spleen and lungs. On the other hand, we found that the Cd:Te ratios in various organs over time differed. Specially, at 1 h post-injection, significant changes in Cd:Te ratios were observed in the liver and kidneys. These findings further indicated that CdTe/ZnS aqQDs degraded in specific organs, especially in the liver and kidneys [26], and implied that the chemical stability of CdTe/ZnS aqQDs *in vitro* can not mimic the biological responses or consequences that occur *in vivo*. As shown in Table 1, CdTe/ZnS aqQDs are stable (the maximal emission and relative fluorescence intensity of CdTe/ZnS aqQDs were not altered) after a 1 h incubation in pH 7.4 buffer solutions.

Metallothionein (MT) is a protein that is inducible by various metallic elements, especially in the liver and kidneys. Cd is a potent inducer of MT, and Cd-MT is stable and stored. MT-bound Cd complexes are believed to be responsible for the long biological half-life of Cd in the body [27,28]. It was previously demonstrated that only free Cd release from QDs can induce MT [26]. In our study, over a period of 28 d, we found that 42.94 and 42.60 ng/g Cd remained in the liver and kidneys, suggesting that either significant disintegration of CdTe/ZnS aqQDs had occurred, or that Cd had been released in the liver and kidneys and had transferred Cd as a Cd-MT complex during this time period. In the kidneys, a trend of increasing tissue concentrations was observed at 28 d (Figure 3A). The reabsorption of Cd indicates that renal elimination of Cd is more difficult [29]. The half-life of Cd in the kidneys appears to be very long. In the present study, very little Cd was distributed in the brain. The brain is a challenging organ for drug delivery because the blood brain barrier (BBB) functions as a gatekeeper guarding the body from exogenous substances. Small QDs may be transferred through a space of 20 nm that separates the capillary endothelium from the astrocytes, or QDs may interact with the receptors located at the BBB [30].

How the stable covalent bonds of QDs were broken biologically in the tissue remains unknown and requires further investigation. If the dissociation of Cd from QDs is closely associated with the toxicity of QDs in the tissues, knowing how to actually define the stability of QDs *in vivo* may inspire the development of new measures to inhibit or lessen such degradation, allowing QDs to remain in use medically, but in a much safer manner.

## Conclusions

The chemical fate of QDs *in vivo* is of great importance. The degradation of QDs, and consequent release of free Cd, can contribute to the overall toxicity of QDs. While QDs in biological systems have been traced using either fluorescent imaging methods or Cd tracking (ICP-MS) analyses, none of these analytical techniques or methods was actually able to define the chemical fate of QDs in *vivo*. By measuring blood kinetic parameters, the biodistributions of Cd and Te in CdTe/ZnS aqQDs in mice, and the Cd:Te ratios in tissues, we clearly demonstrated that QDs are not as stable or biologically inert as they were once widely believed to be. *In vitro*, QDs are chemically stable and do not elicit the same biological responses or consequences as they do *in vivo*. Therefore, measuring Cd using ICP-AES or ICP-MS in order to quantify QDs in the plasma, organs, and excretion samples may not be accurate. New approaches to studying the fate of QDs *in vivo* should be considered. In summary, we believe that, although the present study was a relatively simple study, it provided very important information relative to the chemical fate and biological safety of QDs. We hope that our findings will inspire the manufacture of a new generation of QDs that are just as effective, but are also safer.

## Materials and methods

### Characteristics of CdTe/ZnS aqQDs

The CdTe/ZnS aqQDs used in our experiments were prepared by Zhejiang University. The CdTe/ZnS aqQDs had a CdTe core and a ZnS shell. Thioglycolic acid (TGA) was used as a stabilizer. The molar ratio of Cd^2+^:HTe^-^:TGA was fixed at 3:1:3 (Cd:Te = 3:1), Zn^2+^:Cd^2+^ was fixed at 1:1 (Zn:Cd = 1:1). Glutathione (GSH) was used as both a capping reagent and a sulfur source for the *in situ* growth of the ZnS shell on the CdTe core of the QDs. The pH value of the latter solution was fixed at 8.3. Prior to use in our experiments, the CdTe/ZnS aqQDs stock solutions were centrifuged at 650 × g for 15 min at room temperature to remove large aggregates. The supernatants were then dialyzed for 4 h across a 10 kDa cellulose membrane (Sigma) against a 0.1% solution of thioglycolate (sodium salt, Sigma) at pH 8.3 to remove any free Cd, Te, Zn or other small molecules from the solutions [31-33]. The stock solutions were then further dialysed for 2 h against distilled water (pH 8.3) to remove unbound thioglycolate. The size distribution and surface characteristics of the CdTe/ZnS aqQDs were analyzed using AFM (Nano Scope 3D, Veeco, USA). Meanwhile, their fluorescence spectra, peak wavelengths and fluorescence intensity were measured using a fluorescence spectrometer (RF-5301, Shimadzu, Japan). The concentrations of Cd, Te and Zn in the stock solution were determined as described below. Prior to injecting the mice, the CdTe/ZnS aqQDs solutions were freshly dissolved in a normal sodium medium containing PBS (pH 7.4) and sonicated for 5 min in order to allow the CdTe/ZnS aqQDs particles to disperse evenly throughout the solution. The final concentrations of the solutions were adjusted to 50 nmol/ml (calculated based on the molar mass of Cd).

### Stability of CdTe/ZnS aqQDs *in vitro*

The PLQYs of CdTe/ZnS aqQDs *in situ* (pH 8.3) were measured relative to Rhodamine 6G (Fluka) in ethanol over a period of 80 days. In addition, we mimicked the pH of blood (pH 7.4) in order to investigate the stability of CdTe/ZnS aqQDs *in vitro*. CdTe/ZnS aqQDs (50 nmol/ml) were dissolved in PBS solutions (pH 7.4) at 37°C and added to 10 kDa dialysis tubes. The dialysis tubes were placed in polypropylene beakers containing PBS. The fluorescence spectra and relative fluorescent intensities of the solutions in the tubes were measured using a fluorescence spectrometer 15 min, 30 min, 1 h, 6 h, 24 h, 2 d and 3 d after the tubes were placed in the beakers. The concentrations of Cd, Te and Zn in the filtrate were quantitatively measured using ICP-MS (7500ce, Agilent, USA). The lowest standard (1 ng/ml) on the calibration curve at which the analyte peak was identifiable and reproducible with a precision of less than 20% was designated as the lower limit of quantification. Calibration plots for Cd, Te and Zn were obtained by injecting a series of standard solutions (1, 5, 10, 50, and 100 ng/ml in 2% HNO_3_, flow rate: 1.0 ml/min) into the ICP-MS. Both Cd:Te ratio and Zn:Cd ratio were then calculated based on the concentrations of Cd, Te and Zn. The results for the PBS solutions were compared to those for the initial CdTe/ZnS aqQDs.

### Animals

Healthy male ICR mice (six weeks old) were purchased from Beijing (Military Medical Science Academy of the People’s Liberation Army). The mice were housed in a ventilated, temperature-controlled, and standardized sterile animal room with a 12 h day/night cycle at the China Capital Medical University. The mice were allowed to acclimate to the animal room for 7 days prior to experimentation. All procedures used in this study were performed in accordance with animal welfare protocols that had been approved by the Capital Medical University Animal Care and Use Committee (approval number 2011-X-072).

### Animal treatment

Mice weighing between 23.8 and 26.8 g were administered the CdTe/ZnS aqQD solution via tail vein injection at a dose of 0.2 μmol CdTe/ZnS aqQDs/kg of body weight [13] (which was calculated based on the molar mass of Cd). The mice in the control group were injected with an equivalent volume of normal saline. Preliminary observations of food intake, fur, behavior, mental status, urine and faeces were conducted daily for each mouse. At predetermined time points (1 min, 15 min, 30 min, 1 h, 6 h, 12 h, 24 h, 3 d, 7 d, 14 d, and 28 d), six mice from each exposed group were anesthetized using isoflurane. Retro-orbital blood was collected into Eppendorf tubes containing heparin (10 μl, 500 IU/ml) and stored at 4°C for further analysis. The mice were then euthanized by cervical dislocation, and the hearts, livers, spleens, lungs, kidneys, and brains were collected. Meanwhile, the control mice were also killed, at predetermined time (7 d, 14 d, and 28 d), strictly according to the procedure through which the exposed mice were treated. The body weight of each mouse and the weights of all organs were recorded. Tissues samples were stored at −80°C.

### Measurement of Cd and Te concentrations using ICP-MS

Blood (200 μl) and portions of the excised organs (0.1 − 0.5 g), which had been rinsed with sterile saline to remove any remaining traces of blood, were soaked in nitric acid (HNO_3_, 67%, MOS grade) in a glass beaker overnight. In addition, a 50 ng/ml indium (^115^In) salt solution was added as an internal standard to ensure the accuracy and precision of the technique. To digest the samples, the beakers were heated to 110–120°C in a mixture of HNO_3_ and H_2_O_2_ (volume ratio 4:1) until the solution was colorless and clear. H_2_O_2_ was used to eliminate nitrogen oxide vapors. Lastly, the solution volume was fixed at 2 ml using diluted nitric acid (2%) and the solution was passed through a 0.45 μm pore polyvinylidene fluoride membrane syringe filter. The concentrations of Cd and Te in the filtrates were quantitatively measured using ICP-MS. The mean values of Cd and Te in the control mice were deducted as the background for the blood and organs (heart, liver, spleen, lungs, kidneys, and brain) (Additional file 2: Table S2). Additionally, the Cd:Te ratios in administrative mice were calculated. The *in vivo* results were compared to that of initial CdTe/ZnS aqQDs.

### Kinetic analyses and statistical analyses

Kinetic analyses of Cd and Te in the blood were carried out using Drug and Statistics (DAS) software version 2.0 (Mathematical Pharmacology Professional Committee of China, Shanghai, China). Kinetic parameters were calculated, including the apparent volume of distribution (Vd), AUC, CL, as well as the distribution half-life (*t*_*1*/*2α*_) and *t*_*1*/*2β*_. The kinetic parameters were estimated separately for each animal. The data are expressed as the mean ± SD. t-testing of the data was performed using the Statistical Package for Social Sciences version 13.0 (SPSS13.0). p values < 0.01 or 0.05 were considered to be significant.

## Abbreviations

QDs: Quantum dots; PLQY: Photoluminescent quantum yield; Cd: Cadmium; Te: Tellurium; Zn: Zinc; AFM: Atomic force microscopy; ICP-MS: Inductively coupled plasma-mass spectrometry; ICP-AES: Inductively coupled plasma-atomic emission spectrometer; ESCs: Embryonic stem cells; TEM: Transmission electron microscopy; MT: Metallothionein; BBB: Blood brain barrier; TGA: Thioglycolic acid; GSH: Glutathione; PBS: Phosphate buffered saline; In: Indium; Vd: Apparent volume of distribution; AUC: Area under the blood concentration-time profiles; CL: Clearance; t1/2α: Distribution half-life; t1/2β: Elimination half-life; DAS: Drug and statistics.

## Competing interests

The authors declare that they have no competing financial interests.

## Authors’ contributions

NL supervised the animal experiments and participated in writing and editing the manuscript. YM and SHH synthesized the QDs. YC, HBS and QX are responsible for data and analysis. MMW, HW and YBL performed the experiments, as well as undertaking the ICP-MS analysis. ZWS and PLH are responsible for the study design and writing of the manuscript. All the authors read and approved the final manuscript.

## Supplementary Material

Additional file 1: Table S1 Tissue weights of the control and CdTe/ZnS aqQDs exposure groups. ICR mice were injected via tail vein with CdTe/ZnS aqQDs (0.2 μmol/kg). At predetermined time points (7 d, 14 d, and 28 d) after dosing, the body weight of each mouse and the weights of all organs were recorded. Compared with respective controls, the tissue weights in mice exposed to CdTe/ZnS aqQDs did not vary significantly (*P* >0.05). Click here for file

Additional file 2: Table S2 Concentrations of Cd and Te of control mice. The detection limits for Cd and Te are 0.1 ppb (ng/ml). Click here for file
