# Supplementary material for: Degradation of aqueous synthesized CdTe/ZnS quantum dots in mice: differential blood kinetics and biodistribution of cadmium and tellurium
Source: Part Fibre Toxicol. 2013 Aug 6;10:37. doi: 10.1186/1743-8977-10-37 (PMC3750282; doi:10.1186/1743-8977-10-37)
Supplement: Additional file 1: Table S1 — Tissue weights of the control and CdTe/ZnS aqQDs exposure groups. ICR mice were injected via tail vein with CdTe/ZnS aqQDs (0.2 μmol/kg). At predetermined time points (7 d, 14 d, and 28 d) after dosing, the body weight of each mouse and the weights of all organs were recorded. Compared with respective controls, the tissue weights in mice exposed to CdTe/ZnS aqQDs did not vary significantly (P >0.05). [file 1743-8977-10-37-S1.pdf]

Table S1 Tissue weights of the control and CdTe/ZnS aqQDs exposure groups

Tissue	7 d		14 d		28 d	
	**Control (g)**	**Exposed (g)**	**Control (g)**	**Exposed (g)**	**Control (g)**	**Exposed (g)**
body	28.45 ±1.20	28.10 ±1.46	31.33 ±1.51	31.50 ±1.25	36.58 ±1.38	36.30 ±1.11
heart	0.15 ±0.03	0.15 ±0.02	0.16 ±0.02	0.16 ±0.03	0.17 ±0.04	0.18 ±0.02
liver	1.85 ±0.05	1.85 ±0.05	1.86 ±0.09	1.85 ±0.07	1.92 ±0.08	1.94 ±0.06
spleen	0.13 ±0.03	0.13 ±0.03	0.13 ±0.02	0.13 ±0.02	0.14 ±0.01	0.14 ±0.07
lungs	0.21 ±0.03	0.21 ±0.03	0.24 ±0.02	0.23 ±0.02	0.26 ±0.03	0.26 ±0.05
kidneys	0.43 ±0.04	0.44 ±0.05	0.57 ±0.04	0.56 ±0.11	0.61 ±0.09	0.63 ±0.06
brain	0.43 ±0.02	0.45 ±0.02	0.43 ±0.02	0.41 ±0.04	0.43 ±0.04	0.44 ±0.05

ICR mice were injected via tail vein with CdTe/ZnS aqQDs (0.2 μmol/kg). At predetermined time points (7 d, 14 d, and 28 d) after dosing, the body weight of each mouse and the weights of all organs were recorded. Compared with respective controls, the tissue weights in mice exposed to CdTe/ZnS aqQDs did not vary significantly ($P > 0.05$).

All data are represented as the mean ±SD, n = 6.
