# Supplementary material for: Degradation of aqueous synthesized CdTe/ZnS quantum dots in mice: differential blood kinetics and biodistribution of cadmium and tellurium
Source: Part Fibre Toxicol. 2013 Aug 6;10:37. doi: 10.1186/1743-8977-10-37 (PMC3750282; doi:10.1186/1743-8977-10-37)
Supplement: Additional file 2: Table S2 — Concentrations of Cd and Te of control mice. The detection limits for Cd and Te are 0.1 ppb (ng/ml). [file 1743-8977-10-37-S2.pdf]

Table S2 Concentrations of Cd and Te of control mice

Tissue	Cd (ppb)	Te (ppb)
blood	0.26 ± 0.08	ND
heart	ND	ND
liver	0.60 ± 0.11	ND
spleen	ND	ND
lungs	ND	ND
kidneys	0.46 ± 0.18	ND
brain	ND	ND

ND, not detectable. The detection limits for Cd and Te are 0.1 ppb (ng/ml).

All data are represented as the mean ± SD, n = 6.
